# Mediterranean Dietary Pattern Adherence Modify the Association between FTO Genetic Variations and Obesity Phenotypes

**DOI:** 10.3390/nu9101064

**Published:** 2017-09-26

**Authors:** Firoozeh Hosseini-Esfahani, Gelareh Koochakpoor, Maryam S. Daneshpour, Bahareh Sedaghati-khayat, Parvin Mirmiran, Fereidoun Azizi

**Affiliations:** 1 Nutrition and Endocrine Research Centre, Research Institute for Endocrine Sciences, Shahid Beheshti University of Medical Sciences, 1985717413 Tehran, Iran; f.hosseini@sbmu.ac.ir; 2 Maragheh University of Medical Sciences, 9415969788 Maragheh, Iran; koochakpoorglareh@gmail.com; 3 Cellular Molecular and Endocrine Research Centre, Research Institute for Endocrine Sciences, Shahid Beheshti University of Medical Sciences, 1985717413 Tehran, Iran; daneshpour1388@gmail.com (M.S.D.); b.s.khayat@gmail.com (B.S.-k.); 4 Faculty of Nutrition Sciences and Food Technology, National Nutrition and Food Technology Research Institute, Shahid Beheshti University of Medical Sciences, 1981619573 Tehran, Iran; 5 Endocrine Research Centre, Research Institute for Endocrine Sciences, Shahid Beheshti University of Medical Sciences, 1985717413 Tehran, Iran

**Keywords:** Mediterranean, dietary pattern, FTO polymorphisms, interaction, obesity

## Abstract

There is increasing interest of which dietary patterns can modify the association of fat mass and obesity associated (FTO) variants with obesity. This study was aimed at investigating the interaction of the Mediterranean dietary pattern (Med Diet) with FTO polymorphisms in relation to obesity phenotypes. Subjects of this nested case-control study were selected from the Tehran Lipid and Glucose Study participants. Each case was individually matched with a normal weight control (*n* = 1254). Selected polymorphisms (rs1421085, rs1121980, rs17817449, rs8050136, rs9939973, and rs3751812) were genotyped. Genetic risk score (GRS) were calculated using the weighted method. The Mediterranean dietary score (MDS) was computed. Individuals with minor allele carriers of rs9939973, rs8050136, rs1781749, and rs3751812 had lower risk of obesity when they had higher MDS, compared to wild-type homozygote genotype carriers. The obesity risk was decreased across quartiles of MDS in participants with high GRS (OR: 1, 0.8, 0.79, 0.67) compared to individuals with low GRS (OR: 1.33, 1.06, 0.97, 1.12) (Pinteraction < 0.05). No significant interaction between the GRS and MDS on abdominal obesity was found. A higher Med Diet adherence was associated with lower obesity risk in subjects with more genetic predisposition to obesity, compared to those with lower adherence to the Med Diet and lower GRS.

## 1. Introduction

The obesity epidemic worldwide is fast increasing and affecting individuals of all ages, races, and both genders. It is positively correlated with a series of metabolic abnormalities, including diabetes, hypertension, cancer, and cardiovascular diseases. Obesity is caused by genetic susceptibilities and environmental factors, such as overconsumption of energy and a sedentary lifestyle [1,2,3]. 

Genome-wide association studies (GWAS) have identified several common genetic variants associated with obesity. Of these, fat mass and the obesity-associated gene (FTO) locus was found to be consistently associated with obesity traits in several populations [4,5], which could increase body mass index (BMI) by 0.22–0.66 per risk allele [6]. However, there is increasing interest in ascertaining whether lifestyle factors modify the association of FTO variants and obesity; this could better provide insight into the role of diet/environmental factors in the pathogenesis of obesity [7]. Previous studies have reported that consumption of unhealthy and energy dense food groups, including fried foods and sugar sweetened beverages, could interact with genetic make-up in relation to obesity [8,9], suggesting that a healthy diet and lifestyle could attenuate, at least partly, the risk of obesity attributed to genetic susceptibility [5,7]. However, a meta-analysis did not support the interaction between total energy or macro-nutrient intakes and the FTO genetic variant of rs9939609 in relation to obesity [10]. 

Over the past decade, studying posteriori dietary patterns (e.g., healthy/prudent, western/unhealthy, and traditional dietary patterns) and their relation to obesity has been paid more attention than a single food or dietary component [11,12]. A higher adherence to the Mediterranean dietary pattern (Med Diet) using Mediterranean diet scores (MDS) was associated with a decrease in obesity, regardless of FTO risk alleles [13,14,15]. 

Data on the interaction of FTO polymorphisms with dietary patterns are rare in Asians and Middle-Eastern populations [5], which is why the aim of the current study was to investigate whether the Med Diet could interact with FTO gene polymorphisms (rs1121980, rs1421085, rs9939973, rs8050136, rs17817449, and rs3751812) in isolation or in a combined-form genetic risk score (GRS) in relation to obesity phenotypes among participants of the Tehran Lipid and Glucose Study (TLGS). Identifying those environmental interactions is needed for establishing targeted preventive approaches in individuals with greater genetic susceptibility to obesity.

## 2. Materials and Methods

Subjects of this nested case-control study were selected from among participants of the TLGS, a large-scale, community-based, prospective study being performed on a sample of residents of District 13 of Tehran, the capital of Iran. The first phase of the TLGS was conducted from 1999 to 2001 on 15,005 subjects, aged ≥3 years, and follow-up examinations have been conducted every three years (2002–2005, 2006–2008, 2008–2011, and 2011–2014) to identify newly developed diseases. Details of this ongoing cohort study have been published elsewhere [16,17]. 

Of 11,001 and 9807 individuals aged ≥18 years who participated in baseline and second follow-up surveys, respectively, 1813 subjects were excluded because they were evaluated as obese (BMI ≥ 30 kg/m^2^) at either baseline or the second follow-up survey. In the current study, 1000 cases were randomly selected among the participants who developed obesity in the third (*n* = 528), the fourth (*n* = 416), and the fifth (*n* = 286) phases. Individuals with a history of weight loss or gain >5 kg in the last six months, those who were pregnant and lactating, or those who had taken drugs that affect weight were excluded from the study, leaving 880 cases to be included in the study. Each of these 880 cases was individually pair matched by age (±5 years) and sex with a random control from a population with normal weight at the time that the corresponding case developed obesity. Cases/controls lacking DNA purification in the range of 1.7 < A260/A280 < 2.0, and those whose reported energy intakes divided by the predicted energy intake did not qualify for the ±3 SD (standard deviation) range, were excluded, so that data of 627 pairs of persons with obesity and their matched controls (1254 persons) were ultimately analyzed (Supplementary Figure S1).

Written informed consent was obtained from all participants. The investigation was carried out according to the rules of the Declaration of Helsinki. The study protocol was approved by the ethics committee of the Research Institute for Endocrine Sciences, Shahid Beheshti University of Medical Sciences, Tehran, Iran (grant No. 840; registration code: ISRCTN15898185 DOI 10.1186/ISRCTN15898185)

### 2.1. Measurements

Dietary intake was assessed using a valid and reliable 168-item semi-quantitative food frequency questionnaire (FFQ) to assess the usual food intakes of individuals during the 12 months before the examination [18,19]. The consumption frequency of each food item on a daily, weekly, or monthly basis was converted to daily intakes, and the portion sizes were then converted to grams using measuring cups and spoons. The Iranian food composition table (FCT) is incomplete [20]; therefore, we used the United States Department of Agriculture (USDA) FCT to analyze foods [21]. However, the Iranian FCT was used for some national foods and beverages when these were not listed in the USDA FCT.

The MDS was computed according to Trichopoulou et al. [22], based on the following eight components: Legumes, vegetables, nuts and fruits, fish, and cereals intake; dietary ratio of monounsaturated fatty acid (MUFA) to saturated fatty acid (SFA); intake of dairy products, mostly in the form of cheese or yogurt and intakes of meat and poultry. The energy adjusted of each food component was calculated using the energy density method (gram per 1000 kcal) for determining the MDS. The sex-specific median intake of the eight food components of the typical Med Diet in the population was considered as the cutoff; a value of 1 was assigned to a high intake (≥median) of each of the desirable components, including fruits, nuts, vegetables, legumes, cereals, and fish, or to a low intake (<median) of each of the undesirable foods, i.e., meat and dairy products. Moreover, a value of 0 was assigned for individuals whose consumption was at or above the median for undesirable foods or below the median for desirable foods. For fat intake, the ratio of daily consumption (in grams) of MUFA to SFA was used; a value of 1 was assigned if this ratio was greater than the sex-specific median value and a value of zero was assigned for consumption less than the median. Alcohol consumption is not common in the Iranian population due to religious beliefs and its estimation cannot be precisely conducted in Iran; therefore, alcohol consumption was not considered to be a food component. We then summed up the points for all eight items to calculate the MDS. Thus, the total MDS ranged from 0 (no adherence) to 8 (maximal adherence) [23,24]. 

The body weight of each participant was measured to the nearest 100 g using digital scales while the subjects were minimally clothed and not wearing shoes. Height was measured to the nearest 0.5 cm with a tape measure while the subjects were in a standing position, with their shoulders in a normal alignment and with shoes removed. Circumferences were measured to the nearest millimeter using a flexible tape. Waist circumference (WC) was taken at the end of normal expiration, over light clothing, with the upstretched tape measure positioned at the level of umbilicus, without exerting any pressure on the body surface; measurements were recorded to the nearest 0.1 cm [25]. Hip circumference was measured at the level of maximal protrusion of the gluteal muscles. Waist to hip ratios (WHRs) was calculated as WC (cm) divided by hip circumference (cm) [26]. 

Physical activity level was assessed with high reliability and relatively moderate validity using the Persian translated modifiable activity questionnaire (MAQ). The frequency and time spent on light, moderate, hard, and very hard intensity activities, according to the list of common activities of daily life over the past year, were obtained; and activity data were transformed into metabolic equivalent hours per week (METs/h/week) [27,28]. 

### 2.2. Genotyping

We selected six single-nucleotide polymorphisms (SNPs) within the region of the FTO gene, based on published literature and on the validated GWAS catalog (The NHGRI-EBI Catalog of Published Genome-Wide Association Studies [29] and the Phenotype-Genotype Integrator [30], taking into account minor allele frequency (MAF) > 0.2 and *p* value < 10^−7^. The selected SNPs were associated with dietary intake or obesity phenotypes [4,31,32,33,34,35].

Genomic DNA was extracted from peripheral blood using a standard proteinase K, salting-out method. Six SNPs (rs1421085, rs1121980, rs17817449, rs8050136, rs9939973, and rs3751812) were selected through the NCBI site, as follows: our tetra-primer refractory mutation system-(T-ARMS) assay with different inner allele specific primers was used to produce allele-specific polymerase chain reaction (PCR) products. The two outer primers produced a PCR product that was used as an internal control for the reaction. For all six SNPs, the PCR reaction (Thermal Cycler, Corbett Life Science, Sydney, Australia) was optimized in a 12.5 µL total volume containing 1.5 µL DNA template, 6.25 µL Master Mix containing MgCl_2_, Smart Taq polymerase (CinnaGene Co., Tehran, Iran), and 0.1% BSA (TaKaRa, Kusatsu, Japan), 2 µL primer (outer and inner), and 2.75 µL water. The PCR products were separated by size using agarose gel electrophoresis; each genotype generated a specific band. Accuracy of the results was confirmed by direct sequencing of 10% of each sample using the outer primers. 

### 2.3. Obesity GRS Calculation

GRS was calculated based on the six SNPs using the weighted method [8,9]. Each SNP was recoded as 0, 1, or 2 according to the number of risk alleles (BMI increasing alleles), and each SNP was weighted by its relative effect size (odds ratio) derived from the previously reported meta-analysis or original data. We then calculated the GRS using the following equation:

GRS = (OR1 × SNP1 + OR2 × SNP2 + … + ORn × SNPn) × (*n*/sum of the ORs), where OR is the odds ratio of each individual SNP on BMI, as derived from previous literature (original and reported meta-analyses) [4,32,36], *n* is 6, and sum of the ORs is 8.18 in the current analysis. The GRS ranged from 0 to 12, and each point of the GRS corresponded to each single risk allele.

### 2.4. Definitions

Obesity was defined as a BMI ≥ 30 kg/m^2^, and a BMI between 18.5 and 24.9 classified a person as having a normal weight; WC ≥ 95 cm for both genders, as well as WHR ≥ 0.8 in men and ≥ 0.9 in women, were considered as indicators of abdominal obesity [25,26,37]. 

### 2.5. Statistical Analysis

The descriptive analysis consisted of a comparison of qualitative and quantitative variables between cases and controls using the chi square and Student’s *t* test, respectively; the genotype and allele frequencies for the analyzed polymorphisms were obtained using Power-Marker software (Bioinformatics Research Center Campus Box 7566 North Carolina State University Raleigh, NC 27695-7566, USA). Pearson’s chi-square statistic test was used to calculate the Hardy-Weinberg equilibrium.

Conditional logistic regression was used to estimate the interactions of SNPs and GRS with quartiles of MDS (mean Q1: 1.63, Q2: 3.56, Q3: 5.00, Q4: 6.25) in relation to obesity, after adjustment for educational level (≤14 and >14 years). Two likelihood scores were obtained by performing this statistical analysis, with and without the interaction terms; the *p* value for interaction was determined by performing the likelihood ratio test.

Conditional logistic regression was used to generate odds ratios (ORs) for obesity for individuals as carriers or non-carriers of minor alleles of each SNP across quartiles of MDS. The lowest quartile of MDS and the homozygote group with a major allele were examined as the reference group. Participants were divided into two groups based on the median GRS. Unconditional logistic regression was performed to estimate the interactions of SNPs and GRS with quartiles of MDS in relation to abdominal obesity. All ORs were adjusted for variables proven to be associated with obesity, including age, gender, educational level, smoking status (current, ex-smoker, or never smoked), physical activity (low, moderate, and high) and energy intake; *p* value for trend across the quartiles of dietary fiber was determined using logistic regression, with the median of each quartile of MDS as a continuous variable. Data were analyzed using the STATA (statistics/data analysis v.12.0) or SPSS (Statistical Package for Social Sciences, Version 20.0; Inc., IBM, New York, NY, USA).

## 3. Results

The mean ages of participants were 34.1 ± 11 in men and 34.9 ± 11 in women. Percentage of participants with high educational level (≥14 years) was significantly higher in individuals with normal BMI. Additionally, obese individuals (cases) had higher WC at baseline than controls. Energy and macronutrient intakes did not differ in cases and controls (Table 1).

The allele and genotype frequency of the two groups are shown in Table 2. Genotype frequencies were in Hardy-Weinberg equilibrium in the total population, and did not differ between the two groups. The median of GRS among participants was 6. 

Interactions of MDS and FTO SNPs in relation to obesity are shown in Table 3. MDS modulated the association of FTO SNPs with obesity. Individuals with minor allele carriers of rs9939973, rs8050136, rs1781749, and rs3751812 had lower risk of obesity when they had higher MDS, compared to wild-type homozygote genotype carriers. No significant interactions for obesity risk were found between FTO SNPs rs1121980 and rs1421085 and MDS. 

Individuals with the highest MDS and one copy of minor allele variants; rs8050136, rs1781749, and rs3751812, had lower risk of abdominal obesity compared to those also with the highest MDS but with no copies of the minor allele. The risk of abdominal obesity did not differ between genotypes of rs1121980, rs1421085, and rs9939973 in quartiles of MDS and no interactions were observed (Table 4).

There were significant interactions between the MDS and FTO SNPs rs8050136, rs1781749, and rs3751812 in relation to risk of high WHR, which decreased in participants with one or two minor allele carriers while the MDS increased compared to those homozygotes for the major alleles. We observed no interaction between FTO SNPs rs1121980, rs1421085, and rs9939973 and MDS in relation to high WHR risk (Table 5).

The association of GRS of FTO variants with a risk of obesity phenotypes across quartiles of MDS is shown in Table 6. The obesity risk was decreased across quartiles of MDS in participants with high GRS (OR: 1, 0.8, 0.79, 0.67; *p* for trend = 0.001), compared to individuals with low GRS (OR: 1.33, 1.06, 0.97, 1.12; *p* for trend = 0.33) (*p* for interaction < 0.05). We found no significant interaction between the GRS and MDS on abdominal obesity and high WHR risk. 

## 4. Discussion

In this nested case-control study of men and women, we found that greater adherence to the Med Diet was associated with lower risk of obesity in subjects with more genetic risk alleles of FTO variants compared to subjects with lower adherence to the Med Diet and lower genetic susceptibility to obesity. These findings suggest that persons with more adherences to Med Diet may be more susceptible to the beneficial effects of Med Diet. 

Our study showed significant interaction between FTO variants and MDS in relation to obesity phenotypes. Previous studies on FTO gene-diet interaction have focused more on FTO rs9939609 and its interaction with macro-nutrients in relation to obesity [10]. This is the first study that combined multiple FTO genetic variants in a Middle Eastern population. Moreover adherence to the overall dietary pattern such as the Med Diet is more important than the effect of specific nutrients as the former the cumulative effects of healthful foods or nutrients are considered [14,15,38]. 

The interaction of FTO variant rs1421085 and diet score (composed of 12 different variables) in relation to BMI was reported in Young et al.’s study and the combined estimate was a 0.3% change in BMI per SD of the diet score per FTO risk allele [7]. In an Asian Indian population, carbohydrate and fiber intake modulated the association of FTO SNPs rs8050136 and rs11076023 with obesity traits [5]. Previous studies have examined modifying effect of the Med Diet on the association of genetic variants with obesity traits and they found no significant interaction between the Med Diet and FTO SNP rs9939609 in relation to obesity or anthropometric changes [13,14,15,39]; the only significant gene-diet interaction observed was between MDS and TCF7L2 rs7903146 in relation to weight gain [15]. Homozygous subjects for the risk allele of rs9939609 had higher or lower BMI or WC depending on greater or lesser adherence to the Med Diet, which can be clinically significant [39]. Good adherence to the Med Diet weakened the association of FTO rs9939609 polymorphism and type 2 diabetes [40]. 

Over the past few decades, the Med Diet has been identified as a healthy dietary pattern which may play an important role in reducing obesity. Evidence of Med Diet adherence in a non-Mediterranean population and its relation to obesity is limited [23]. In a Japanese population, higher adherence to the Med Diet was associated with a lower prevalence of obesity [41]. However, in an Iranian population no such association was found in a cross-sectional study [42]; also, in a longitudinal study conducted on Iranian adults, greater adherence to the Med Diet did not predict lower incidence of metabolic syndrome and its components [24]. Most previous studies of Mediterranean populations showed that high adherence to the Med Diet was associated with a lower increase in abdominal obesity in the long term [15,38,43]. 

The Med Diet consist of high consumption of olive oil daily, which is the main source of fat, and is characterized by high intakes of vegetables, fruit, pulses/legumes, whole grains, nuts, and seeds, moderate consumption of seafood, fermented dairy products (cheese and yogurt), poultry, eggs, and red wine [44], and low consumption of red meat, meat products, and sweets. The healthy benefits of the Med Diet have been attributed to antioxidants and anti-inflammatory properties of its food items. Antioxidants have the capacity to modulate gene and protein expression. Previous nutrigenomic studies have shown that the Med Diet has a protective role on the expression of pro-atherosclerotic genes [43,45]. 

The strengths of our study include its prospective design with long-term follow-up, pair matching cases with controls by age and sex, extensive adjustment for potential confounders, use of a priori-defined dietary pattern analysis to better detect the association of the overall dietary composition. Selection new cases of obesity reduced the possibility of any dietary behavior changes. Furthermore, use of a valid and reliable FFQ and physical activity questionnaire are the other strengths of our study. However, measurement errors of dietary factors are inevitable.

However, our study does have its limitations; the population was small and highly homogeneous, because the study was performed only on sample of residents of district 13 of Tehran. The small sample size reduced the statistical power for possible interactions with abdominal obesity. There are other SNPs which are associated with obesity phenotypes, whereas our study included only six SNPs of the FTO gene. The effect of unknown or unmeasured confounding factors, such as parental obesity, household income, sleep duration, and occupational status cannot be excluded. Moreover, BMI is less precise and underestimates the prevalence of adiposity when compared to direct methods of measuring fat mass [46,47,48]. 

The dietary patterns or composition of foods of Iranian population as a non-Mediterranean country are very different from populations living in Mediterranean countries due to variations in quantity of food intakes (e.g., fish, olive oil, and *n*-3 PUFA, alcohol), and differences in processing and preparation of foods. Moreover, food preferences in each food group may also affect eating patterns in each meal. Finally, scoring participants according to the Med Diet might have variations in different populations and, consequently, the findings of the current study may not be applicable to Mediterranean countries; however, these results can be generalized to non-Mediterranean countries, such as those in the Middle East and North Africa.

## 5. Conclusions

Our findings suggest that Med Diet adherence can be useful for prevention or treatment of obesity phenotypes in subjects with FTO risk alleles. This study could be replicated in other ethnic and demographic populations to confirm our findings.

Regarding individual genetic make-up and dietary pattern as a major environmental factor shift us toward personalized nutritional advice; as individuals should be genotyped and given the dietary recommendation based on their joint associations of dietary pattern and genotypes. Additionally, nutrition interventions for preventing or treatment of obesity need to be promoted and targeted according to subgroups with FTO or other SNP risk alleles.

## Figures and Tables

**Table 1:** Characteristics of the study population in cases and controls ^a^ (Tehran Lipid and Glucose Study).

	Normal BMI ^b^ (*n* = 627)		Obese ^c^ (*n* = 627)	
	Mean	SD	Mean	SD
Baseline Age (year)				
Men	34.01	11	34.25	11
Women	34.82	11	34.91	10
Current smokers (%)	16.7		13.9	
Low physical activity (%)	40.2		41.3	
Education level ≥ 14 years (%)	27.3		20.4 ^d^	
Baseline BMI (kg/m^2^)	21.54		25.03 ^d^	
Baseline WC (cm)	73.54	16	82.25 ^d^	23
Energy intake (kcal/day)	2379	978	2535	1055
Carbohydrate (% of energy)	58.07	6	58.08	6
Total fiber intake (gram per 1000 kcal)	18.06	6	18.57	6
Protein intake (% of energy)	14.46	3	14.44	2
Total fat (% of energy)	30.9	6	30.7	6
Saturated fat (% of energy)	10.25	3	10.07	2
MUFA (% of energy)	10.51	2	10.41	2
PUFA (% of energy)	6.35	2	6.32	2
MDS	4.00	1.44	4.00	1.45

BMI: body mass index, WC: waist circumference. MUFA, mono-unsaturated fatty acids, PUFA, poly-unsaturated fatty acids, MDS: Mediterranean dietary score. ^a^ values are mean unless otherwise listed. ^b^ Normal BMI is from 18.5 up to 25 kg/m^2^, ^c^ BMI ≥ 30 kg/m^2^, ^d^ *p* < 0.05

**Table 2:** Allele and genotype frequency of FTO SNPs in cases (obese) and controls (non-obese): Tehran Lipid and Glucose Study.

	Normal BMI ^a^ (*n* = 627)	Obese ^b^ (*n* = 627)
Allele frequency rs1121980	A:38 (463) ^c^	A: 39 (474)
	G: 62 (755)	G: 61 (752)
Genotype frequency rs1121980	AA: 15 (92)	AA: 13 (77)
	GA: 46 (279)	GA: 52 (320)
	GG: 39 (238)	GG: 35 (216)
Allele frequency rs1421085	C: 37 (458)	C: 36 (446)
	T: 63 (770)	T: 64 (792)
Genotype frequency rs1421085	CC:14 (87)	CC:12 (72)
	TC: 44 (272)	TC: 51 (314)
	TT: 42 (260)	TT: 37 (228)
Allele frequency rs9939973	A: 37.9 (468)	A: 38.2 (469)
	G: 62.1 (768)	G: 61.8 (759)
Genotype frequency rs9939973	AA: 15 (93)	AA: 12.2 (76)
	AG: 45.6 (282)	AG: 52 (319)
	GG: 39.3 (243)	GG: 35.8 (220)
Allele frequency rs8050136	A:34 (417)	A: 35 (435)
	G: 66 (823)	G: 65 (795)
Genotype frequency rs8050136	AA: 13 (78)	AA: 11 (66)
	GA: 42 (261)	GA: 49 (303)
	GG: 45 (281)	GG: 40 (246)
Allele frequency rs17817449	G: 33 (413)	G: 36 (444)
	T: 67 (832)	T: 64 (802)
Genotype frequency 17817449	GG: 12 (75)	GG: 11 (68)
	TG: 42 (264)	TG: 49 (308)
	TT: 46 (284)	TT: 40 (247)
Allele frequency rs3751812	G: 67 (829)	G: 65 (799)
	T: 33 (405)	T: 35 (427)
Genotype frequency rs3751812	GG: 47 (290)	GG: 40 (247)
	GT: 40 (249)	GT: 50 (305)
	TT: 13 (78)	TT: 10 (61)

^a^ BMI: 18.5–25 kg/m^2^, ^b^ BMI ≥ 30 kg/m^2^, ^c^ % (*n*); All allele frequencies were in Hardy-Weinberg equilibrium (*p* > 0.2), except the allele frequency of rs9939973 in obese participants (*p* = 0.01).

**Table 3:** Adjusted ORs (95%CI) for obesity according to quartiles of MDS by FTO SNP genotypes ^a^ (Tehran Lipid and Glucose Study).

	MDS				*p* for Trend	*p* for Interaction
	Q1	Q2	Q3	Q4		
rs1121980						0.65
CC	1	0.8	0.77	0.76	0.4	
		(0.44–1.45)	(0.38–1.54)	(0.37–1.56)		
CT+TT	1.55	0.81	1.05	1.1	0.67	
	(0.55–1.98)	(0.46–1.43)	(0.57–1.92)	(0.57–2.14)		
rs1421085						0.58
TT	1	0.76	0.75	0.69	0.31	
		(0.43–1.33)	(0.38–1.45)	(0.35–1.37)		
CT+CC	1.05	0.84	0.97	1.19	0.56	
	(0.56–1.94)	(0.49–1.45)	(0.54–1.75)	(0.63–2.26)		
rs9939973						0.05
GG	1	0.87	0.87	0.76	0.53	
		(0.48–1.56)	(0.44–1.71)	(0.38–1.54)		
AG+AA	1.11	1.08	0.88	0.55	0.007	
	(0.59–2.07)	(0.60–1.96)	(0.51–1.54)	(0.21–1.41)		
rs8050136						
CC	1	0.74	1	0.77	0.31	0.03
		(0.42–1.28)	(0.56–1.78)	(0.41–1.44)		
AC+AA	1.22	0.98	0.84	0.64	0.004	
	(0.65–2.29)	(0.54–1.80)	(0.50–1.42)	0.33–1.23))		
rs1781749						
TT	1	0.79	0.77	0.72	0.38	0.03
		(0.46–1.36)	(0.46–1.36)	(0.46–1.36)		
TG+GG	1.3	1.02	0.92	0.27	0.004	
	(0.69–2.43)	(0.57–1.81)	(0.54–1.55)	(0.15–0.97)		
rs3751812						
GG	1	0.79	0.8	1.06	0.36	0.04
		(0.46–1.35)	(0.43–1.49)	(0.59–1.89)		
TG+TT	1.33	1.12	0.97	0.67	0.005	
	(0.71–2.20)	(0.61–2.05)	(0.58–1.64)	(0.34–1.28)		

MDS: Mediterranean dietary score, OR: odds ratio, Q: quartiles of MDS, FTO: fat mass and obesity-associated gene, SNP: single-nucleotide polymorphism. ^a^ ORs (95%CI) were calculated using a conditional logistic regression model, adjusted for education level. Participants were classified (eight groups) according to quartiles of MDS and genotypes. The highest quartile of MDS and homozygote genotype of major alleles were used as the reference group. Obesity defined as BMI ≥ 30 kg/m^2^.

**Table 4:** Adjusted ORs (95%CI) ^a^ for abdominal obesity according to quartiles of MDS by FTO SNP genotypes (Tehran Lipid and Glucose Study).

	MDS				*p* for Trend	*p* for Interaction
	Q1	Q2	Q3	Q4		
rs1121980						0.36
CC	1	0.72	0.88	0.61	0.35	
		(0.38–1.38)	(0.41–1.88)	(0.28–1.34)		
CT+TT	0.8	0.84	0.86	1.11	0.34	
	(0.41–1.63)	(0.45–1.58)	(0.44–1.68)	(0.54–2.25)		
rs1421085						0.3
TT	1	0.77	0.97	0.63	0.39	
		(0.41–1.44)	(0.46–2.01)	(0.29–1.36)		
CT+CC	0.92	0.96	0.91	1.25	0.34	
	(0.4–1.46)	(0.52–1.76)	(0.47–1.74)	(0.62–2.51)		
rs9939973						0.39
GG	1	0.76	0.95	0.64	0.43	
		(0.40–1.44)	(0.45–2.02)	(0.29–1.40)		
AG+AA	0.84	0.89	0.89	1.14	0.4	
	(0.42–1.70)	(0.48–1.65)	(0.46–1.71)	(0.56–2.30)		
rs8050136						
CC	1	0.84	1.04	0.97	0.45	0.02
		(0.46–1.53)	(0.52–2.09)	(0.49–1.92)		
AC+AA	1.35	1	0.93	0.65	0.004	
	(0.68–2.67)	(0.55–1.81)	(0.49–1.76)	(0.31–1.37)		
rs1781749						
TT	1	0.89	1.08	0.7	0.52	0.02
		(0.49–1.61)	(0.54–2.17)	(0.33–1.46)		
TG+GG	1.45	1.06	1.04	0.51	0.004	
	(0.73–2.85)	(0.59–1.91)	(0.53–2.04)	(0.12–1.68)		
rs3751812						
GG	1	0.85	1.1	0.67	0.86	0.01
		(0.47–1.54)	(0.55–2.19)	(0.32–1.41		
TG+TT	1.39	1.09	1.01	0.44	0.007	
	(0.70–2.75)	(0.61–1.96)	(0.51–1.99)	(0.09–1.87)		

MDS: Mediterranean dietary score, OR: odds ratio, Q: quartiles of MDS, FTO: fat mass and obesity-associated gene, SNP: single-nucleotide polymorphism. ^a^ ORs (95%CI) were calculated using conditional logistic regression model, adjusted for education level, age, gender, smoking status, physical activity, and energy intake. Participants were classified (eight groups) according to quartiles of MDS and genotypes. The highest quartile of MDS and homozygote genotype of major allele were used as the reference group. WC > 95 cm for both genders were defined as the abdominal obesity according to the Iranian cutoff.

**Table 5:** Adjusted ORs (95%CI) ^a^ for high waist to hip ratio according to quartiles of MDS by FTO SNP genotypes (Tehran Lipid and Glucose Study).

	MDS				*p* for Trend	*p* for Interaction
	Q1	Q2	Q3	Q4		
rs1121980						0.69
CC	1	0.67	0.8	0.98	0.87	
		(0.31–1.46)	(0.31–2.03)	(0.36–2.64)		
CT+TT	1.03	0.8	1.5	0.98	0.66	
	(0.44–2.43)	(0.37–1.69)	(0.63–3.56)	(0.40–2.40)		
rs1421085						0.67
TT	1	0.71	0.83	1.17	0.69	
		(0.34–1.48)	(0.24–2.01)	(0.43–2.89)		
CT+CC	1.2	0.88	1.57	1.03	0.87	
	(0.52–2.73)	(0.43–1.78)	(0.69–3.06)	(0.43–2.43)		
rs9939973						0.74
GG	1	0.6	0.79	0.93	0.88	
		(0.28–1.31)	(0.31–2.01)	(0.34–2.50)		
AG+AA	0.95	0.79	1.35	0.94	0.64	
	(0.40–2.21)	(0.37–1.67)	(0.57–3.18)	(0.38–2.30)		
rs8050136						
CC	1	0.61	0.79	1.05	0.47	0.04
		(0.30–1.26)	(0.33–1.87)	(0.41–2.67)		
AC+AA	1.45	0.98	0.83	0.8	0.001	
	(0.62–3.37)	(0.43–2.21)	(0.41–1.69)	(0.36–2.05)		
rs1781749						
TT	1	0.68	0.8	1.12	0.73	0.03
		(0.33–1.36)	(0.35–1.84)	(0.44–2.80)		
TG+GG	1.61	1.09	0.95	0.81	0.007	
	(0.70–3.69)	(0.48–2.45)	(0.41–2.21)	(0.44–1.77)		
rs3751812						
GG	1	0.6	0.8	1.05	0.75	0.03
		(0.29–1.22)	(0.34–1.87)	(0.40–2.65)		
TG+TT	1.52	0.91	0.81	0.61	0.006	
	(0.65–3.55)	(0.40–2.06)	(0.41–1.65)	(0.22–1.47)		

MDS: Mediterranean dietary score, OR: odds ratio, Q: Quartiles of MDS, FTO: fat mass and obesity-associated gene, SNP: single-nucleotide polymorphism. ^a^ ORs (95%CI) were calculated by using a conditional logistic regression model, adjusted for education level, age, gender, smoking status, physical activity, and energy intake. Participants were classified (eight groups) according to quartiles of MDS and genotypes. The highest quartile of MDS and homozygote genotype of major allele were used as the reference group. WHR ≥ 0.8 in men and ≥0.9 in women, were considered as indicators of high waist to hip ratio.

**Table 6:** Adjusted ORs (95%CI) ^a^ for obesity and abdominal obesity according to quartiles of MDS by GRS ^b^.

	MDS				*p* for Trend	*p* for Interaction
	Q1	Q2	Q3	Q4		
Obesity						0.04
GRS ≥ 6	1	0.8	0.79	0.67	0.001	
		(0.43–1.49)	(0.46–1.35)	(0.34–1.29)		
GRS < 6	1.33	1.06	0.97	1.12	0.33	
	(0.71–2.49)	(0.59–1.89)	(0.58–1.64)	(0.61–2.05)		
Abdominal obesity						0.29
GRS ≥ 6	1	1.01	0.97	1.37	0.37	
		(0.62–1.65)	(0.55–1.68)	(0.75–2.52)		
GRS < 6	0.95	0.8	1.02	0.65	0.48	
	(0.48–1.87)	(0.48–1.32)	(0.55–1.90)	(0.34–1.28)		
High WHR						0.37
GRS ≥ 6	1	0.8	1.57	0.77	0.93	
		(0.43–1.51)	(0.71–3.47)	(0.35–1.71)		
GRS < 6	0.96	0.59	0.79	1.07	0.65	
	(0.42–2.20)	(0.31–1.10)	(0.36–1.75)	(0.44–2.61)		

MDS: Mediterranean dietary score, GRS: genetic risk score, OR: odds ratio, Q: quartiles of MDS, WHR: Waist to hip ratio. ^a^ ORs (95%CI) were calculated using a conditional logistic regression model, adjusted for education level. ^b^ GRS was calculated on the basis of the six selected single nucleotide polymorphisms of the fat mass and obesity-associated gene (FTO) using a weighted method. Participants were classified (eight groups) according to quartiles of MDS and GRS. The highest quartile of MDS and GRS < 6 (the median of GRS) were used as the reference group.

## Supplementary Materials

The following are available online at www.mdpi.com/2072-6643/9/10/1064/s1.

## References

[1] Jafari-Adli S., Jouyandeh Z., Qorbani M., Soroush A., Larijani B., Hasani-Ranjbar S. (2014). Prevalence of obesity and overweight in adults and children in Iran; a systematic review. J. Diabetes Metab. Disord..

[2] Ng M., Fleming T., Robinson M., Thomson B., Graetz N., Margono C., Mullany E.C., Biryukov S., Abbafati C., Abera S.F. (2014). Global, regional, and national prevalence of overweight and obesity in children and adults during 1980–2013: A systematic analysis for the Global Burden of Disease Study 2013. Lancet.

[3] Park S.L., Cheng I., Pendergrass S.A., Kucharska-Newton A.M., Lim U., Ambite J.L., Caberto C.P., Monroe K.R., Schumacher F., Hindorff L.A. (2013). Association of the FTO obesity risk variant rs8050136 with percentage of energy intake from fat in multiple racial/ethnic populations: The PAGE study. Am. J. Epidemiol..

[4] Peng S., Zhu Y., Xu F., Ren X., Li X., Lai M. (2011). FTO gene polymorphisms and obesity risk: A meta-analysis. BMC Med..

[5] Vimaleswaran K.S., Bodhini D., Lakshmipriya N., Ramya K., Anjana R.M., Sudha V., Lovegrove J.A., Kinra S., Mohan V., Radha V. (2016). Interaction between FTO gene variants and lifestyle factors on metabolic traits in an Asian Indian population. Nutr. Metab. Lond..

[6] Vimaleswaran K.S., Angquist L., Hansen R.D., van der A.D., Bouatia-Naji N., Holst C., Tjonneland A., Overvad K., Jakobsen M.U., Boeing H. (2012). Association between FTO variant and change in body weight and its interaction with dietary factors: The DiOGenes study. Obesity.

[7] Young A.I., Wauthier F., Donnelly P. (2016). Multiple novel gene-by-environment interactions modify the effect of FTO variants on body mass index. Nat. Commun..

[8] Qi Q., Chu A.Y., Kang J.H., Huang J., Rose L.M., Jensen M.K., Liang L., Curhan G.C., Pasquale L.R., Wiggs J.L. (2014). Fried food consumption, genetic risk, and body mass index: Gene-diet interaction analysis in three US cohort studies. BMJ.

[9] Qi Q., Chu A.Y., Kang J.H., Jensen M.K., Curhan G.C., Pasquale L.R., Ridker P.M., Hunter D.J., Willett W.C., Rimm E.B. (2012). Sugar-sweetened beverages and genetic risk of obesity. N. Engl. J. Med..

[10] Qi Q., Kilpelainen T.O., Downer M.K., Tanaka T., Smith C.E., Sluijs I., Sonestedt E., Chu A.Y., Renstrom F., Lin X. (2014). FTO genetic variants, dietary intake and body mass index: Insights from 177,330 individuals. Hum. Mol. Genet..

[11] Mu M., Xu L.F., Hu D., Wu J., Bai M.J. (2017). Dietary Patterns and Overweight/Obesity: A Review Article. Iran. J. Public Health.

[12] Rezagholizadeh F., Djafarian K., Khosravi S., Shab-Bidar S. (2017). A posteriori healthy dietary patterns may decrease the risk of central obesity: Findings from a systematic review and meta-analysis. Nutr. Res..

[13] Livingstone K.M., Celis-Morales C., Navas-Carretero S., San-Cristobal R., Forster H., O’Donovan C.B., Woolhead C., Marsaux C.F., Macready A.L., Fallaize R. (2016). Fat mass- and obesity-associated genotype, dietary intakes and anthropometric measures in European adults: The Food4Me study. Br. J. Nutr..

[14] Razquin C., Martinez J.A., Martinez-Gonzalez M.A., Bes-Rastrollo M., Fernandez-Crehuet J., Marti A. (2010). A 3-year intervention with a Mediterranean diet modified the association between the rs9939609 gene variant in FTO and body weight changes. Int. J. Obes. Lond..

[15] Roswall N., Angquist L., Ahluwalia T.S., Romaguera D., Larsen S.C., Ostergaard J.N., Halkjaer J., Vimaleswaran K.S., Wareham N.J., Bendinelli B. (2014). Association between Mediterranean and Nordic diet scores and changes in weight and waist circumference: Influence of FTO and TCF7L2 loci. Am. J. Clin. Nutr..

[16] Azizi F., Rahmani M., Emami H., Mirmiran P., Hajipour R., Madjid M., Ghanbili J., Ghanbarian A., Mehrabi Y., Saadat N. (2002). Cardiovascular risk factors in an Iranian urban population: Tehran lipid and glucose study (phase 1). Soz. Praventivmed..

[17] Azizi F., Ghanbarian A., Momenan A.A., Hadaegh F., Mirmiran P., Hedayati M., Mehrabi Y., Zahedi-Asl S. (2009). Prevention of non-communicable disease in a population in nutrition transition: Tehran Lipid and Glucose Study phase II. Trials.

[18] Esfahani F.H., Asghari G., Mirmiran P., Azizi F. (2010). Reproducibility and relative validity of food group intake in a food frequency questionnaire developed for the Tehran Lipid and Glucose Study. J. Epidemiol..

[19] Mirmiran P., Esfahani F.H., Mehrabi Y., Hedayati M., Azizi F. (2010). Reliability and relative validity of an FFQ for nutrients in the Tehran lipid and glucose study. Public Health Nutr..

[20] Azar M., Sarkisian E. (1980). Food composition table of Iran.

[21] Food Composition Table (FCT) Food and Nutrition Information Center, US Department of Agriculture, 2009. www.nal.usda.gov/fnic/foodcomp.

[22] Trichopoulou A., Costacou T., Bamia C., Trichopoulos D. (2003). Adherence to a Mediterranean diet and survival in a Greek population. N. Engl. J. Med..

[23] Asghari G., Farhadnejad H., Mirmiran P., Dizavi A., Yuzbashian E., Azizi F. (2017). Adherence to the Mediterranean diet is associated with reduced risk of incident chronic kidney diseases among Tehranian adults. Hypertens. Res..

[24] Mirmiran P., Moslehi N., Mahmoudof H., Sadeghi M., Azizi F. (2015). A Longitudinal Study of Adherence to the Mediterranean Dietary Pattern and Metabolic Syndrome in a Non-Mediterranean Population. Int. J. Endocrinol. Metab..

[25] Azizi F., Khalili D., Aghajani H., Esteghamati A., Hosseinpanah F., Delavari A., Larijani B., Mirmiran P., Mehrabi Y., Kelishadi R. (2010). Appropriate waist circumference cut-off points among Iranian adults: The first report of the Iranian National Committee of Obesity. Arch. Iran. Med..

[26] World Health Organization (2008). Waist Circumference and Waist–Hip Ratio: Report of a WHO Expert Consultation.

[27] Kriska A.M., Knowler W.C., LaPorte R.E., Drash A.L., Wing R.R., Blair S.N., Bennett P.H., Kuller L.H. (1990). Development of questionnaire to examine relationship of physical activity and diabetes in Pima Indians. Diabetes Care.

[28] Momenan A.A., Delshad M., Sarbazi N., Rezaei Ghaleh N., Ghanbarian A., Azizi F. (2012). Reliability and validity of the Modifiable Activity Questionnaire (MAQ) in an Iranian urban adult population. Arch. Iran. Med..

[29] GWAS Catalog The NHGRI-EBI Catalog of Published Genome-Wide Association Studies. https://www.ebi.ac.uk/gwas/.

[30] PheGenI, Phenotype-Genotype Integrator. https://www.ncbi.nlm.nih.gov/gap/phegeni.

[31] McCaffery J.M., Papandonatos G.D., Peter I., Huggins G.S., Raynor H.A., Delahanty L.M., Cheskin L.J., Balasubramanyam A., Wagenknecht L.E., Wing R.R. (2012). Obesity susceptibility loci and dietary intake in the Look AHEAD Trial. Am. J. Clin. Nutr..

[32] Lee H.J., Kim I.K., Kang J.H., Ahn Y., Han B.G., Lee J.Y., Song J. (2010). Effects of common FTO gene variants associated with BMI on dietary intake and physical activity in Koreans. Clin. Chim. Acta.

[33] Wing M.R., Ziegler J., Langefeld C.D., Ng M.C., Haffner S.M., Norris J.M., Goodarzi M.O., Bowden D.W. (2009). Analysis of FTO gene variants with measures of obesity and glucose homeostasis in the IRAS Family Study. Hum. Genet..

[34] Moore S.C., Gunter M.J., Daniel C.R., Reddy K.S., George P.S., Yurgalevitch S., Devasenapathy N., Ramakrishnan L., Chatterjee N., Chanock S.J. (2012). Common genetic variants and central adiposity among Asian-Indians. Obesity.

[35] Tanaka T., Ngwa J.S., van Rooij F.J., Zillikens M.C., Wojczynski M.K., Frazier-Wood A.C., Houston D.K., Kanoni S., Lemaitre R.N., Luan J. (2013). Genome-wide meta-analysis of observational studies shows common genetic variants associated with macronutrient intake. Am. J. Clin. Nutr..

[36] Alharbi K.K., Richardson T.G., Khan I.A., Syed R., Mohammed A.K., Boustred C.R., Gaunt T.R., Tamimi W., Al-Daghri N.M., Day I.N. (2014). Influence of adiposity-related genetic markers in a population of saudi arabians where other variables influencing obesity may be reduced. Dis. Markers.

[37] Consultation W.E. (2004). Appropriate body-mass index for Asian populations and its implications for policy and intervention strategies. Lancet.

[38] Funtikova A.N., Benitez-Arciniega A.A., Gomez S.F., Fito M., Elosua R., Schroder H. (2014). Mediterranean diet impact on changes in abdominal fat and 10-year incidence of abdominal obesity in a Spanish population. Br. J. Nutr..

[39] Corella D., Ortega-Azorin C., Sorli J.V., Covas M.I., Carrasco P., Salas-Salvado J., Martinez-Gonzalez M.A., Aros F., Lapetra J., Serra-Majem L. (2012). Statistical and biological gene-lifestyle interactions of MC4R and FTO with diet and physical activity on obesity: New effects on alcohol consumption. PLoS ONE.

[40] Ortega-Azorin C., Sorli J.V., Asensio E.M., Coltell O., Martinez-Gonzalez M.A., Salas-Salvado J., Covas M.I., Aros F., Lapetra J., Serra-Majem L. (2012). Associations of the FTO rs9939609 and the MC4R rs17782313 polymorphisms with type 2 diabetes are modulated by diet, being higher when adherence to the Mediterranean diet pattern is low. Cardiovasc. Diabetol..

[41] Kanauchi M., Kanauchi K. (2016). Development of a Mediterranean diet score adapted to Japan and its relation to obesity risk. Food Nutr. Res..

[42] Asghari G., Mirmiran P., Rashidkhani B., Asghari-Jafarabadi M., Mehran M., Azizi F. (2012). The association between diet quality indices and obesity: Tehran Lipid and Glucose Study. Arch. Iran. Med..

[43] Martinez-Gonzalez M.A., Salas-Salvado J., Estruch R., Corella D., Fito M., Ros E., Predimed I. (2015). Benefits of the Mediterranean Diet: Insights From the PREDIMED Study. Prog. Cardiovasc. Dis..

[44] Davis C., Bryan J., Hodgson J., Murphy K. (2015). Definition of the Mediterranean Diet; a Literature Review. Nutrients.

[45] Fito M., Konstantinidou V. (2016). Nutritional Genomics and the Mediterranean Diet’s Effects on Human Cardiovascular Health. Nutrients.

[46] Frankenfield D.C., Rowe W.A., Cooney R.N., Smith J.S., Becker D. (2001). Limits of body mass index to detect obesity and predict body composition. Nutrition.

[47] Shah N.R., Braverman E.R. (2012). Measuring adiposity in patients: The utility of body mass index (BMI), percent body fat, and leptin. PLoS ONE.

[48] Romero-Corral A., Somers V.K., Sierra-Johnson J., Thomas R.J., Collazo-Clavell M.L., Korinek J., Allison T.G., Batsis J.A., Sert-Kuniyoshi F.H., Lopez-Jimenez F. (2008). Accuracy of body mass index in diagnosing obesity in the adult general population. Int. J. Obes. Lond..

