# Supplementary material for: Mediterranean Dietary Pattern Adherence Modify the Association between FTO Genetic Variations and Obesity Phenotypes
Source: Nutrients. 2017 Sep 26;9(10):1064. doi: 10.3390/nu9101064 (PMC5691681; doi:10.3390/nu9101064)
Supplement: Supplementary file 1 [file nutrients-09-01064-s001.pdf]

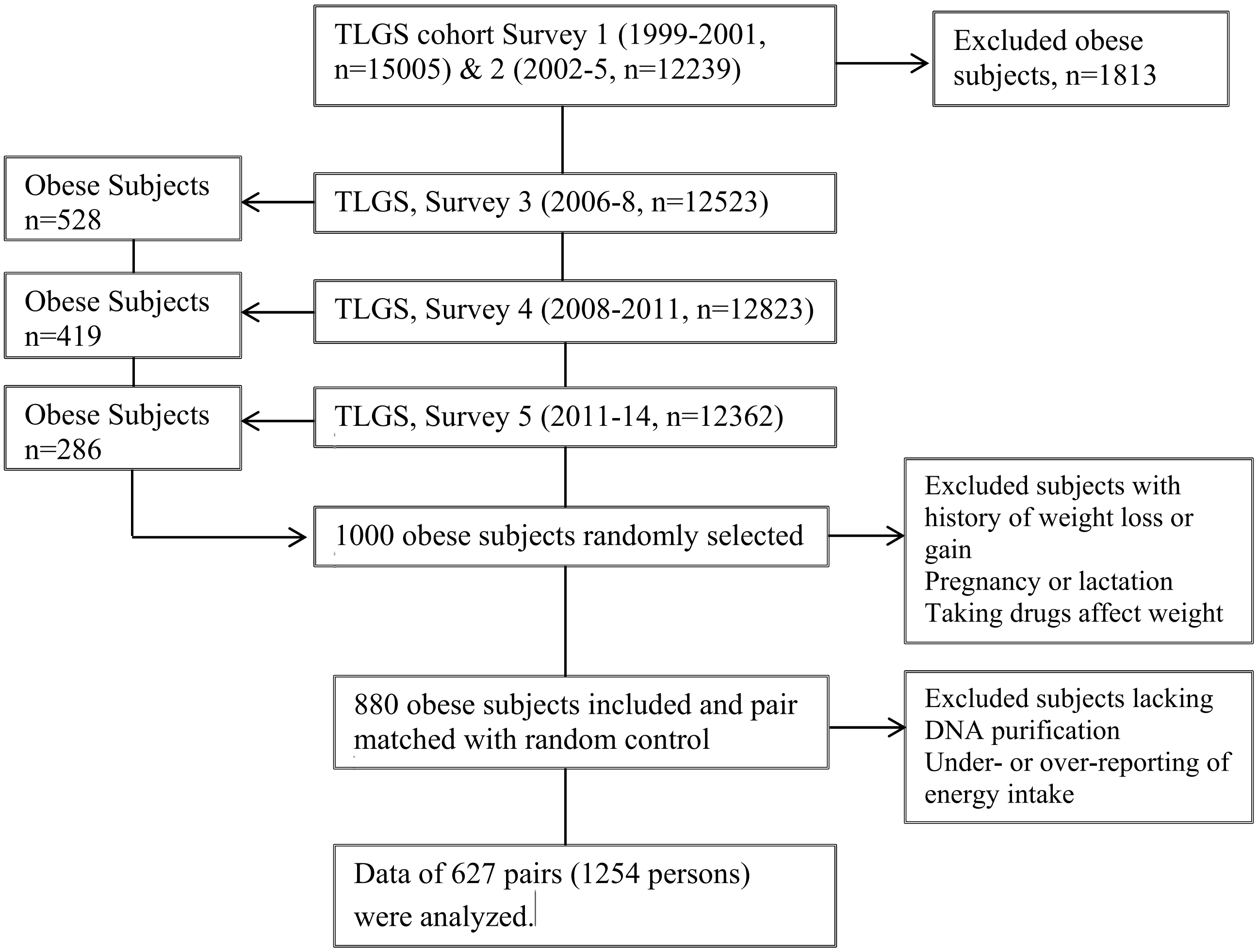

Figure. Flowchart of study participants

TLGS: Tehran Lipid and Glucose Study
MetS: Metabolic Syndrome
